# An Impedance-Loaded Orthogonal Frequency-Coded SAW Sensor for Passive Wireless Sensor Networks

**DOI:** 10.3390/s20071876

**Published:** 2020-03-28

**Authors:** Xuan Dai, Lili Fang, Chuanfang Zhang, Houjun Sun

**Affiliations:** School of Information and Electronics, Beijing Institute of Technology, Beijing 100081, China; 2702002011@bit.edu.cn (X.D.); zcf@bit.edu.cn (C.Z.); sunhoujun@bit.edu.cn (H.S.)

**Keywords:** passive wireless sensor, impedance-loaded, surface acoustic wave, orthogonal frequency coded, structural health monitoring

## Abstract

A passive wireless impedance-loaded orthogonal frequency-coded (OFC) surface acoustic wave (SAW) sensor for wireless sensor networks was proposed in this paper. One of the chips on OFC SAW tag is connected to an external sensor, which could cause a phase shift in the time response of the corresponding part on the SAW device. The phase shift corresponds to the sensed quantity, which could be temperature, strain, vibration, pressure, etc. The OFC SAW tag is isolated by a proper package from the direct effect of the measurand on the device’s response which could avoid the multiple measurands coupling. The simultaneous work of multiple sensors is guaranteed by orthogonal frequency coding. By processing the response based on an extended matched filter algorithm, sensing information of the specific coded OFC device can be extracted from the superimposed response of multiple independent encoded sensors. Compared to previous methods, the proposed method can produce a more flexible passive (battery-free) wireless sensor suitable for large-scale wireless sensor networks. Simulation and experimental results demonstrate the effectiveness of the sensor.

## 1. Introduction

Wireless sensors could be used for structural health monitoring (SHM) systems which are desired on most of the aircraft for dynamic monitoring [1,2,3]. In recent years, researchers show an increasing interest in wireless passive sensor technology [4,5,6,7,8,9,10,11], since it does not need battery supply and is maintenance free. Compared with the traditional wireless sensing technologies [12,13,14], wireless passive sensors could have a long operational life cycle, which is feasible for harsh environment applications.

A spiral inductive loop antenna with a pressure-sensitive capacitive element could realize wireless passive pressure sensing [15]. The applied pressure will change the capacitive value which leads to the resonant frequency shift of the sensor, wireless operation is performed by the near-field electromagnetic coupling between the sensor and an external antenna. However, the working distance of this sensor is extremely short, which is determined by the principle of the near-field electromagnetic coupling.

Surface Acoustic Wave (SAW) sensor is a type of micro-machined passive wireless sensors that have been widely investigated and used for strain, pressure and temperature sensing, etc. [16,17,18,19,20,21,22]. A SAW sensor system consists of two parts: a SAW device connected to an antenna as the sensor transponder which need not battery supply, a local reader unit which is used for interrogation signal transmitting, echoed data receiving and data processing. Since a large time delay could be obtained using a compact-sized SAW device, the clutter of environmental reflection could be separated from the echoed data, hence SAW sensors could work in the harsh outdoor environment.

SAW sensors could be classified into two types: delay line SAW sensors which could reflect the measurand by the time delay of the reflectors and SAW resonators (SAWR) which could reflect the measurand by the variation of the resonant frequency [23]. SAW sensors could also be grouped into one-port SAW sensors and two-port SAW sensors which are also called impedance-loaded SAW sensors. One-port SAW sensors are directly affected by the measurand, impedance-loaded SAW sensors are electrically loaded by a conventional sensor, as a result, indirectly affected by the measurand [24,25,26]. In the impedance-loaded SAW sensors, the SAW device is used as a transponder for device identification, while the measurand is sensed by an external sensor. The impedance variations of the external sensor will accordingly affect the reflection coefficient of the SAW device, which could be extracted from the echoed data.

The principles for separating the transmitted and received signals include time-domain division (TDD), frequency-domain division (FDD) [27]. However, the number of signal processing channels of a sensor using the TDD principle is limited by the physical size of the sensor, and the number of channels using the FDD principle is limited by the IDT bandwidth of the sensor, so they are not suitable for large-scale wireless sensor networks.

To expand the capacity of SAW sensors, an OFC SAW sensor based on the principle of orthogonal frequency-coded division is proposed in [28,29,30]. This sensor could expand the capacity in a limited bandwidth using code diversity. However, this OFC SAW sensor is a one-port sensor which means the response is directly affected by the measurand, so the accuracy could not be guaranteed since sensor response might be sensitive to multiple measurands simultaneously.

A novel impedance-loaded OFC SAW sensor is proposed in this paper. The device identification is accomplished by orthogonal frequency coding, and the measurand is sensed by an external sensor connected to the reflectors on the SAW device. The relationship between the impedance value of the external sensor and the response of the SAW device is investigated, the effect of the external impedance on the orthogonality is simulated, and an extended matched filter algorithm is proposed for the information extraction. Simulation results and experimental results show that the presented impedance-loaded OFC SAW sensor could provide a large capacity for passive wireless sensor networks while avoiding the multiple measurand coupling effects on the SAW device.

## 2. Principle of Impedance-Loaded OFC SAW Sensors

### 2.1. Principle of Orthogonal Frequency Coding

Orthogonal Frequency Coding is a spread-spectrum coding technique. An OFC signal could have both frequency and time diversity which will provide a systematic way of implementing code in a SAW device embodiment [31]. For an OFC signal, 1 bit is divided into M chips, which have the same chip interval $\tau_{chip}$. The time response function $h_{ci}\left(t\right)$ of every chip is as follows:

(1) $$h_{ci}\left(t\right)=cos[2\pi f_{ci}\left(t-i\cdot \tau_{chip}\right)$$

where $f_{ci}$ is the single local carrier frequency of the $i^{th}$ chip.

The time-domain response of an OFC signal could be expressed as:

(2) $$h_{OFC}\left(t\right)=\sum_{i=1}^{M}a_{i}\cdot rect\left(\frac{t-i\cdot \tau_{chip}}{\tau_{chip}}\right)\cdot cos\left[2\pi f_{ci}\left(t-i\cdot \tau_{chip}\right)\right]$$

where $a_{i}$ is the amplitude of the $i^{th}$ chip, and

(3) $$rect\left(x\right)=\left\{\begin{array}{c} 1,\left|x\right|\leq 0.5\\ 0,otherwise \end{array}\right\}$$

According to the orthogonality in orthogonal frequency coding, the product of the local single carrier frequency $f_{ci}$ and time interval $tau_{chip}$ in the chip must be an integer, and the minimum frequency difference between the local single carrier frequency $f_{ci}$ is $1/\tau_{chip}$.

**Figure 1:**
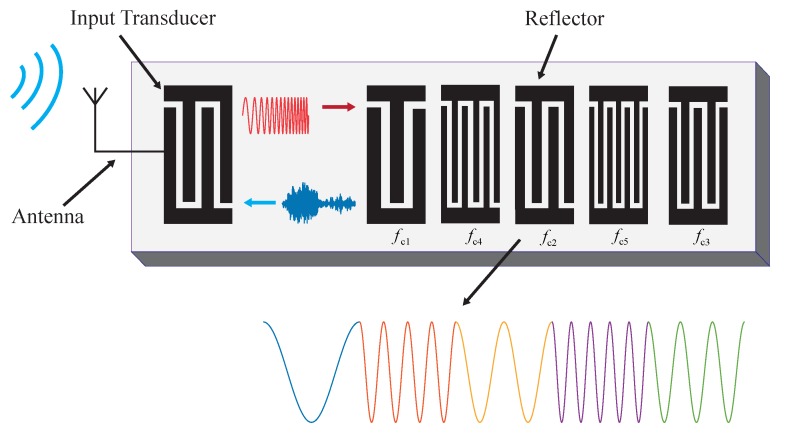
Schematic of a 5-chip wireless OFC SAW tag.

The local single carrier frequency $f_{ci}$ on each chip could be realized on SAW device through the coupling of modes (COM) analysis of the reflectors. A typical schematic of the OFC SAW tags is shown in Figure 1:

In Figure 1, there are five chips with different local single carrier frequency, whose position could be interchanged to construct a new OFC SAW device. The OFC device with different code are orthogonal to each other, and the capacity of coding could be 5!. The code shown in Figure 1 is $f_{c1},f_{c4},f_{c2},f_{c5},f_{c3}$, which can be abbreviated as (14253).

### 2.2. Principle of Impedance-Loaded SAW Devices

**Figure 2:**
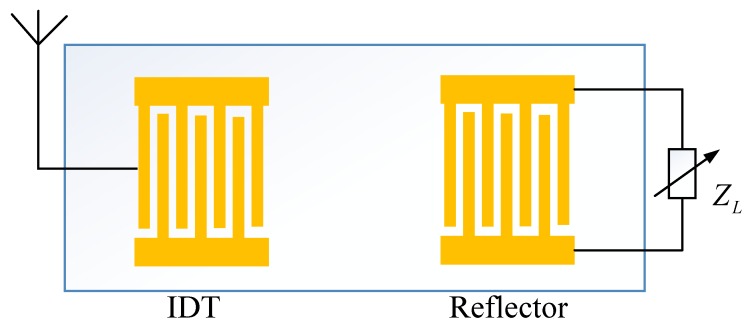
Schematic of an impedance-loaded SAW sensor.

Impedance-loaded SAW sensors use electrically loaded IDT to avoid the coupling effect of multiple measurands caused by the direct measurement of the SAW sensor [23]. A classic schematic of the impedance-loaded SAW sensor is shown in Figure 2:

In Figure 2, the IDT on the left is connected to the antenna, and the reflector on the right is electrically loaded by an external impedance $Z_{L}$, which could be physical, chemical or biological sensors. The external impedance $Z_{L}$ would be changed according to the variation of the quantity to be sensed. Every reflector on the device could be modeled as a device with two acoustic ports and one electrical port in the well-known P-matrix formalism. The total response of the SAW device could be obtained through the P-matrix cascade [32]. The reflective characteristics of the electrically loaded IDT is a function of the external impedance $Z_{L}$, which can be expressed in the form of P-Matrix as [33]:

(4) $$P_{11}\left(Z_{L}\right)=P_{11,sc}+\frac{2\cdot P_{13}^{2}}{P_{33}+\frac{1}{Z_{L}}}$$

where $P_{11}$ is acoustic reflectivity of the electrically loaded reflector, $P_{11,sc}$ is the reflectivity for electrical short, $P_{13}$ is the electro-acoustic transfer coefficient and $P_{33}$ is the input admittance of the electrically loaded reflector. From Equation (4), when the external impedance is capacitive, the effect of the electrically loaded reflector on the reflective characteristics is obvious, while the inductive or resistive impedance has little effect.

When the quantity to be sensed varies, the impedance of the external traditional sensor is changed accordingly, and the reflective characteristics of the IDT reflector connected to it will change as a result. In general, the echoed data reflected by the SAW device which is a response to the interrogation signal transmitted by the interrogator would contain the information of the impedance variation of the external traditional sensor.

## 3. Wireless Passive Impedance-Loaded OFC SAW Sensors

Impedance-loaded SAW sensors could be applied to various physical, chemical and biological quantities measurement by changing the type of the external sensor; however, the identification of different Impedance-loaded SAW sensors could only be achieved by time-domain diversity which is limited by the size of the SAW device, the capacity for multi-sensor operation is small.

Orthogonal Frequency-Coded (OFC) SAW device has a rather large capacity for multi-sensor operation, the number of available codes could be M!. However, the OFC SAW sensor is operated by coupling the measurand with the time delay of the OFC SAW device which might be sensitive to multiple quantities simultaneously. In such an OFC SAW sensor, it is hard to eliminate response changes due to unwanted variables from the total response.

**Figure 3:**
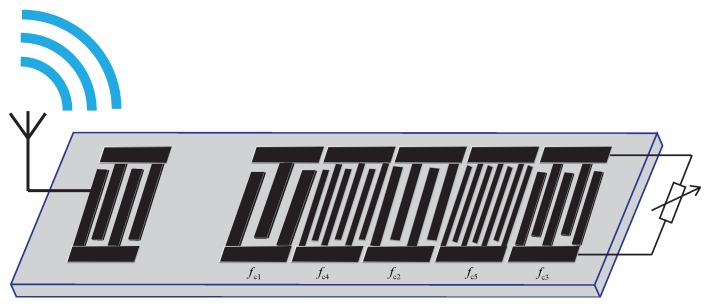
Schematic of an impedance-loaded OFC SAW sensor.

In this paper, the topology of an impedance-loaded Orthogonal Frequency-Coded SAW sensor is presented. The schematic of the proposed sensor is given in Figure 3.

In Figure 3, the measurand is sensed by an external sensor which is connected to the last chip of the OFC SAW device as an external impedance load, the SAW device can be isolated from the variation of the measurand by proper packaging, and the identification between multiple sensors are guaranteed by orthogonality between OFC codes. When the impedance value of the external traditional sensor is changed, the reflectivity factor of the OFC SAW device would be changed according to Equation (4).

### 3.1. Relationship Between Δϕ and the External Impedance

Based on Equation (4), there is a single-value relationship between the external impedance which is connected to the last chip of the OFC SAW device and the $P_{11}$ value which could be translated into $S_{11}$ value in Scattering Matrix. Given a capacitive external impedance *C*, $Z_{L}$ would be $\frac{1}{j\omega C}$. Considering $P_{11,sc}$ is nearly zero for a split-finger IDT, Equation (4) could be written as follows:

(5) $$P_{11}\left(C\right)=\frac{2P_{13}^{2}}{\sqrt{P_{33}^{2}+{\left(\omega C\right)}^{2}}}e^{j\Delta \phi }$$

where ω is the angular frequency.

For the narrowband case, it could be approximated that Δϕ does not change with angular frequency ω variation, so there is an additional phase shift Δϕ in the impulse response of the last chip, the actual impulse response of the impedance-loaded OFC SAW device $h_{IL\_OFC}\left(t\right)$ could be written as follows:

(6) $$\begin{array}{cc} h_{IL\_OFC}\left(t\right)= & \sum_{i=1}^{M-1}a_{i}\cdot rect\left(\frac{t-i\cdot \tau_{chip}}{\tau_{chip}}\right)\cdot cos\left[2\pi f_{ci}\left(t-i\cdot \tau_{chip}\right)\right]+\\ \; & a_{M}\cdot rect\left(\frac{t-M\cdot \tau_{chip}}{\tau_{chip}}\right)\cdot cos\left[2\pi f_{cM}\left(t-M\cdot \tau_{chip}\right)+\Delta \phi \right] \end{array}$$

**Figure 4:**
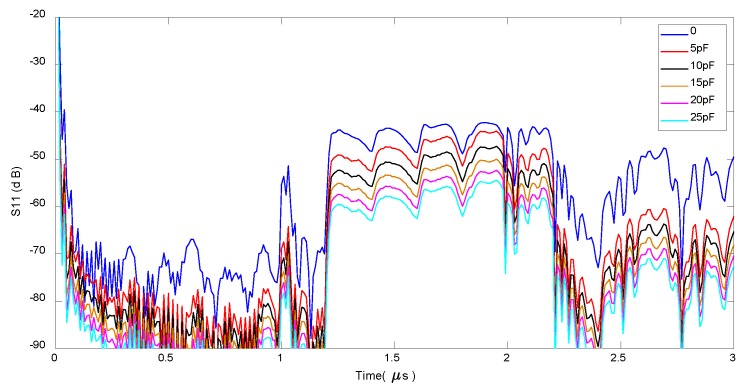
Amplitude response of an impedance-loaded OFC SAW sensor.

**Figure 5:**
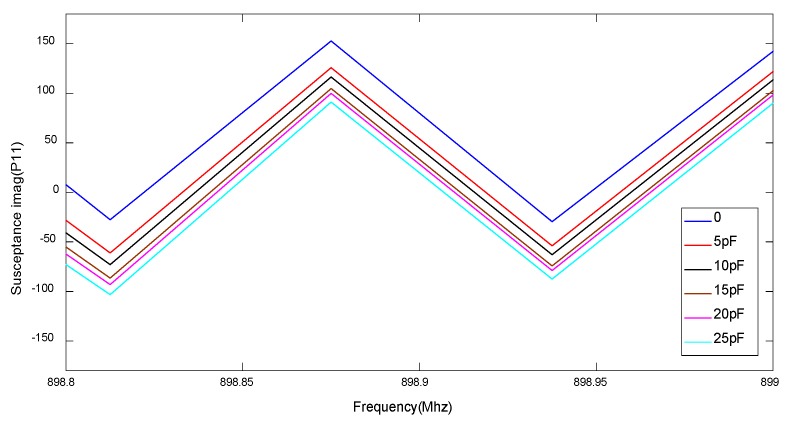
Phase response of an impedance-loaded OFC SAW sensor.

The impedance-loaded OFC SAW device shown in Figure 3 is modeled in MATLAB using COM methods, the impulse response of the device under a series of external impedance loads is simulated, the amplitude of the impulse response and the phase of the impulse response is shown in Figure 4 and Figure 5 respectively. In Figure 4, the amplitude of impulse response decreases when the external capacitive value increases from 0pF to 25pF. In Figure 5, the phase of the impulse response on the last chip of the OFC SAW device changes when the external capacitive value varies.

Since the approximately uniform of the amplitude response for all the five coded reflectors is achieved by apodization which is a cosine weigh to the reflectors, the amplitude response of the total device is quite low, but OFC SAW has an advantage of enhanced signal processing gain which is maintained by the inherent of the spread-spectrum technology. Moreover, the reflectivity of the reflectors could be further improved by embedding the reflector electrodes into the substrate.

### 3.2. Effect of Δϕ on Orthogonality

Based on the orthogonality between different coded SAW device, the response of the specific coded SAW device could be extracted from the superimposition echoed data of the multiple sensors. Since the last chip on the OFC SAW device is slightly deviated from the ideal orthogonal code, the effect of the phase shift value on the orthogonality must be taken into account.

**Figure 6:**
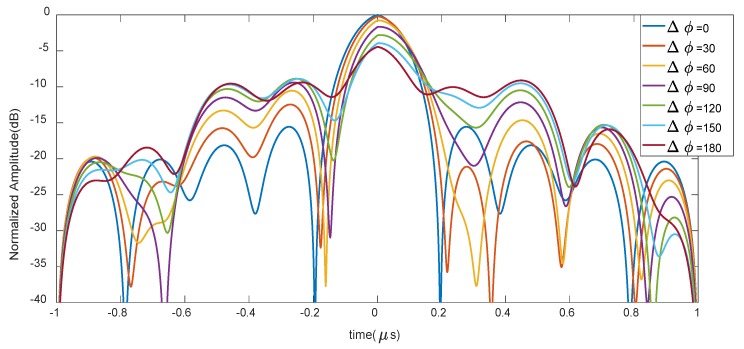
Autocorrelation results of OFC tags as Δϕ varies.

The impulse response $h_{IL\_OFC}\left(t\right)$ with a series of phase shift values Δϕ, could be correlated with a reference function which has the same OFC coding and the phase shift value is set to zero, the correlation result is shown in Figure 6.

From Figure 6, as Δϕ increases, a considerable decrease in the amplitude of the correlation result will occur, and the maximum amplitude reduction is larger than 5 dB.

**Figure 7:**
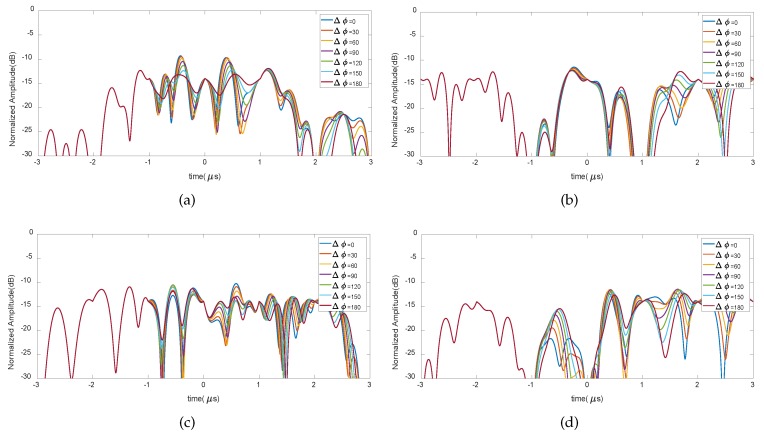
Cross-correlation results of OFC tag (14253) as Δϕ varies. (**a**) with OFC tag (23541); (**b**) with OFC tag (32145); (**c**) with OFC tag (12345); (**d**) with OFC tag (43251).

The impulse response $h_{IL\_OFC}\left(t\right)$ with a series of phase shift values Δϕ, could also be correlated with reference functions with specific orthogonal frequency codes which are consistent with the fabricated ones, and the cross-correlation results are shown in Figure 7.

From Figure 7, the effect of the phase shift value Δϕ has a minus effect on the cross-correlation results. Compared with the autocorrelation results, the normalized cross-correlation results are below 10 dB.

### 3.3. Extended Matched Filter Algorithm for Δϕ Extraction

The traditional matched filter algorithm is not suitable for the response extraction of the specific coded impedance-loaded OFC SAW sensor from the superimposition echoed data of the multiple sensors, because the phase of the last chip slightly deviates from the ideal orthogonal code. From Equation (5), the phase value of the last chip is related to the externally loaded impedance, so it should be extracted to include the measurand. It is our intention that if we constructed a series of reference signals with different phase shift values on the last chip and correlate them with the echoed data, a series of matched filtered responses could be obtained. The maximum peak response will correspond to the reference signal whose phase shift value equals to the phase shift value of the echoed data.

The echoed data reflected from the SAW device $S_{echo}\left(t\right)$ is the convolution of the transmitted signal $s_{t}\left(t\right)$ with the impulse responses of all the SAW devices, it could be expressed as follows:

(7) $$S_{echo}\left(t\right)=S_{echo\_IL1}\left(t\right)+S_{echo\_other}\left(t\right),$$

where $S_{echo\_IL}\left(t\right)$ is the convolution of $s_{t}\left(t\right)$ with the impulse response of the impedance-loaded OFC SAW device $h_{IL1\_OFC}\left(t\right)$, and $S_{echo\_other}\left(t\right)$ is the convolution of $s_{t}\left(t\right)$ with the impulse responses of other orthgonal coded impedance-loaded SAW devices $\sum_{i=2}^{M-1}h_{ILi\_OFC}\left(t\right)$.

(8) $$\begin{array}{cc} \; & S_{echo\_IL1}\left(t\right)=s_{t}\left(t\right)*\left(h_{IL1\_OFC}\left(t\right)\right)\\ \; & S_{echo\_other}=s_{t}\left(t\right)*\sum_{i=2}^{M}h_{ILi\_OFC}\left(t\right) \end{array}$$

Assume the reference signal used for echoed data processing is $S_{ref}\left(t\right)$,

(9) $$S_{ref}\left(t\right)=s_{t}\left(t\right)*h_{ref}^{\prime }\left(t\right),$$

$h_{ref}^{\prime }\left(t\right)$ is the constructed impulse response with a series of phase shift values $\phi_{ref}$ on the last chip,

(10) $$\begin{array}{cc} h_{ref}^{\prime }\left(t\right)= & \sum_{i=1}^{M-1}rect\left(\frac{t-i\cdot \tau_{chip}}{\tau_{chip}}\right)cos\left[2\pi f_{ci}\left(t-i\cdot \tau_{chip}\right)\right]+\\ \; & rect\left(\frac{t-M\cdot \tau_{chip}}{\tau_{chip}}\right)cos\left[2\pi f_{cM}\left(t-M\cdot \tau_{chip}\right)+\phi_{ref}\right] \end{array}$$

The correlation function between $S_{echo}\left(t\right)$ and $S_{ref}\left(t\right)$ could be expressed as

(11) $$r\left(t^{\prime }\right)=\int_{0}^{T}S_{echo}\left(t\right)S_{ref}\left(t+t^{\prime }\right)dt$$

When $S_{ref}\left(t\right)$ is matched with $S_{echo\_IL1}\left(t\right)$, the correlation function $r(t^{\prime })$ could take the maximum amplitude, and $\phi_{ref}$ is equal to Δϕ.

**Figure 8:**
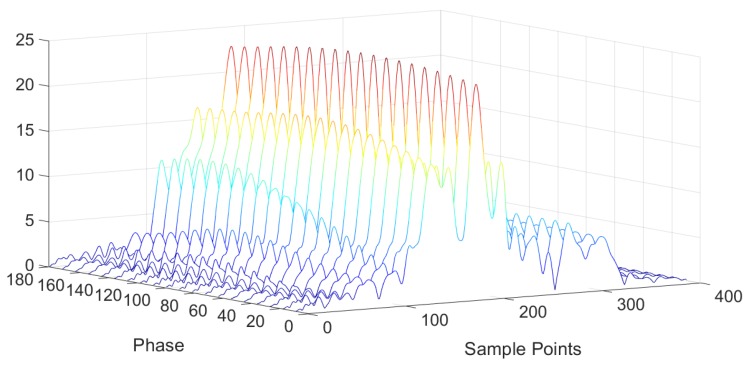
Correlation responses versus phase $\phi_{ref}$ variation.

**Figure 9:**
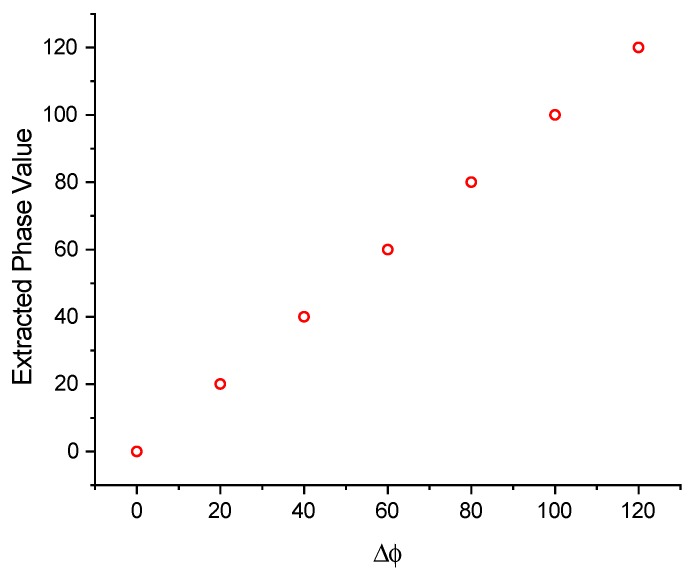
Extracted phase values for different Δϕ.

Assume s(t) is a Dirac function, the echoed data is the mixture of two parts: (1) the impulse response $h_{IL1\_OFC}\left(t\right)$ which has the OFC code (14253); (2) the impulse responses $h_{ILi\_OFC}\left(t\right)$ which have the OFC codes (23541), (32145), (12345) and (43251). the external impedance of the device is taken as 15pF, which is close to the actual situation. the Δϕ value extraction by the extended matched filter algorithm is simulated in Figure 8. Figure 8 is the correlation results for $\Delta \phi =80^{\circ }$ with $S_{ref}\left(t\right)$ where $\phi_{ref}$ varies from 0 to 180 degree. It is shown that the $\phi_{ref}$ value corresponding to the maximum amplitude of correlation is $80^{\circ }$, which is exactly equal to the Δϕ value to be extracted. The extracted phase values based on the extended matched filter algorithm for different Δϕ values are shown in Figure 9. The extracted phase values are in good agreement with the actual Δϕ values.

## 4. Results and Discussion

### 4.1. Impulse Response of the Impedance-Loaded OFC SAW Sensor

**Figure 10:**
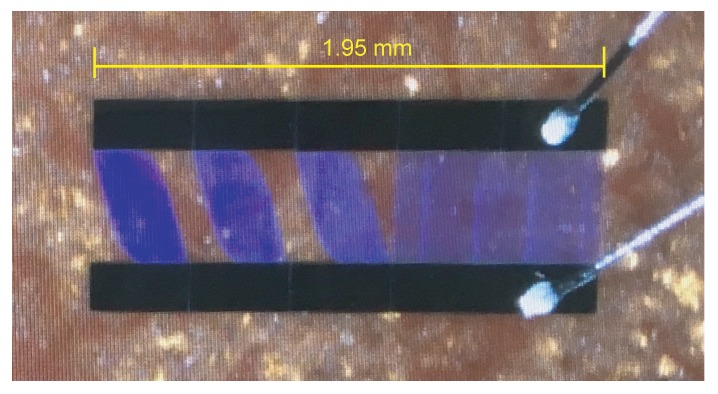
The fabricated impedance-loaded OFC SAW sensor.

A series of impedance-loaded OFC SAW sensors with $128^{\circ }YX-LiNbO3$ substrate material are fabricated. Considering the cost problem, 10 random sets of codes are selected from 120 sets of codes (M = 5) for the SAW devices fabrication. Among the fabricated devices, 5 devices with relatively better insert loss compared with other fabricated devices, i.e., the SAW sensors with the code (12345), (14253), (23541), (32145) and (43251), are selected. The $S_{11}$ values of these sensors are approximate −40∼−50 dB. The schematic of the sensor is shown in Figure 10. The central frequency of the device is 890 MHz and the bandwidth is 32 MHz. The time interval of each chip on the device is 200 ns. The total sensor die dimension is 10 mm × 3 mm. The black areas are bus bars of the SAW device, the blue areas are the apodized reflectors, where the cosine weighed technology is used for controlling the reflection and transmission characteristics of each reflector in an OFC SAW device.

**Figure 11:**
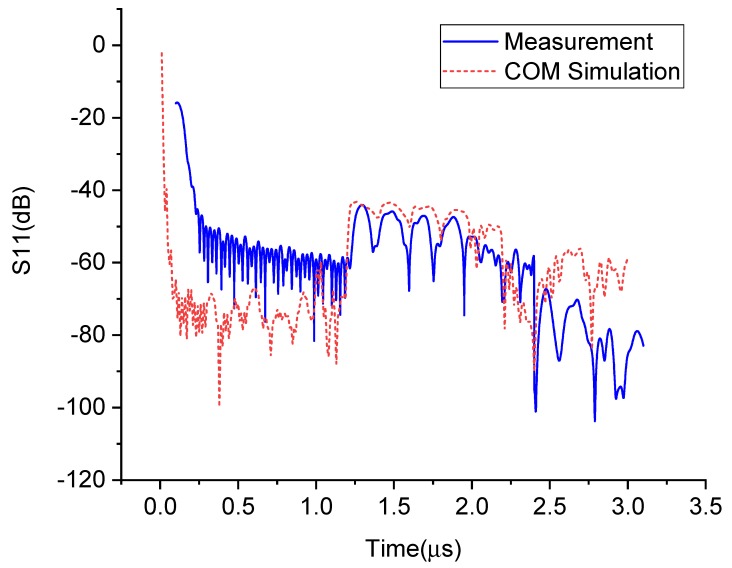
Comparison of the experimental result with the simulation result of the impulse response for an impedance-loaded OFC SAW sensor when impedance value is set to C=0.

A comparison of the experimental result and the simulation result of the impulse response for the sensor with OFC code (12345) when external impedance C=0 is shown in Figure 11, where the experimental result is obtained by Vector Network Analyzer (VNA) and the simulation result is achieved by COM method analyzing. From Figure 11, the responses of the five chips on the OFC SAW device are located in 1.2–2.2 us which is determined by the position of the reflector on the SAW device, and echoes data outside this area could be eliminated by a rectangular window function before further processing, the measurement result is consistent with the simulation one.

### 4.2. Instrumentation and Experimental Setup

**Figure 12:**
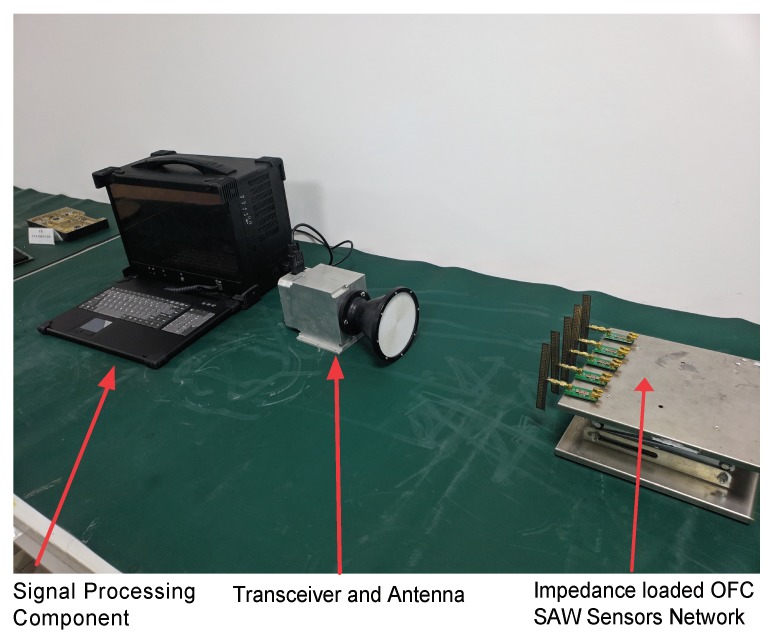
The experimental setup for the impedance-loaded OFC SAW sensors.

The experimental setup is shown in Figure 12. The impedance-loaded OFC SAW sensors with different OFC codes (12345), (14253), (23541), (32145) and (43251) are placed linearly on a test frame, to form multiple passive wireless sensor networks (WSN). The front ends of the sensors are connected to microstrip antennas to receive and transmit the electromagnetic waves sent by the interrogator. The interrogator consists two parts: (a) Antenna and transceiver component, which is used for transmitting interrogator signals and receive the echoed data; (b) Signal processing component, which is used for the measurand information extraction from the echoed data.

**Figure 13:**
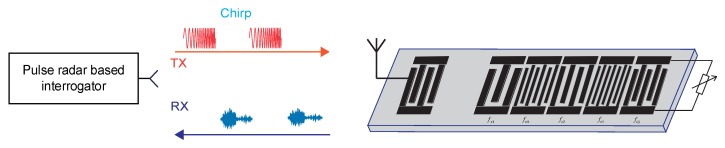
Interrogation with a pulse radar-based reader.

**Figure 14:**
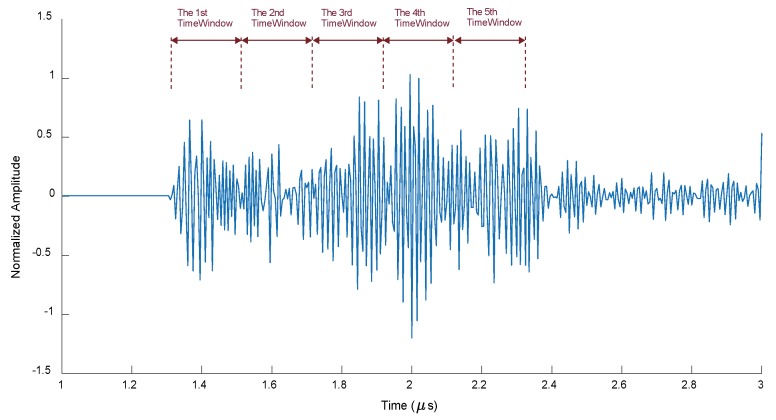
Measured waveform in time domain from multiple impedance-loaded OFC SAW sensors.

A series of chirp signals which are transmitted by the interrogator and received by the impedance-loaded OFC SAW sensors are shown in Figure 13. The signals are reflected by SAW reflectors, the response of the sensor is the convolution of the chirp signals and the impulse response of sensors. Since there are multiple sensors working together, the actual echoed data received by the interrogator is the superimposition of the responses of multiple sensors, which is shown in Figure 14.

**Figure 15:**
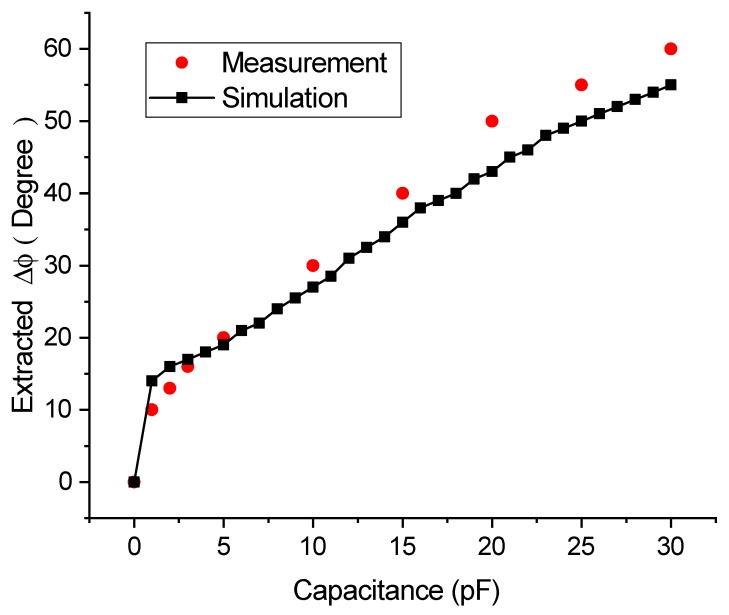
Comparison of experimental and simulation results of relationship between external impedance values and the extracted phase Δϕ for impedance-loaded OFC SAW sensors.

The external impedance is electrically loaded to the SAW sensor with the code (12345). A comparison of experimental and simulation results of the relationship between external impedance values and the phase variations in impedance-loaded OFC SAW sensors is shown in Figure 15. In Figure 15, the red dots are measured results and the black rectangular marks are simulated results. The measured results are close to the simulated ones. However, there are tiny deviations at several points, this is probably caused by the manufacturing error of the sensors.

When the external capacitive value is changed from 0pF to 30pF, the relative phase shift of the sensor response which is extracted by the extended matched filter algorithm from the superimposed response of the five sensors will be changed from 0 to 60 degree accordingly.

### 4.3. Analysis

From Figure 15, in a passive wireless sensor networks with 5 different impedance-loaded OFC SAW sensors, the phase shift value of the specified OFC SAW sensor Δϕ could be correctly extracted, and there is a single-value relationship between this phase shift value and the impedance value of the external sensors. The sensor proposed in this paper has the ability to sense a variety of measurand, since the type of external sensors can be arbitrary, as long as the measurand could cause changes in the impedance of the external sensor. The sensor proposed here could be employed in a large-capacity sensor network, the coding capacity is up to M! where M is the number of chips on a single sensor.

The previous OFC SAW sensors have large coding capacity for multi-sensors operation while the problem of multiple measurands coupling is hard to resolve [28,29,30], since the principle of these sensors is based on the effect of the measurand on the velocity of the surface acoustic wave. In this article, the proposed impedance-loaded OFC SAW sensor is packaged properly so it could be isolated from the measurands, so the problem of multiple measurand coupling could be avoided. The measurand is measured by the external sensors, which would cause a phase shift on the response of the SAW device. Since the phase shift value has a minus effect on orthogonality, the large capacity of OFC coding is kept for this sensor. Compared with previously presented passive wireless OFC SAW sensors [28,29,30,31], this proposed sensor could avoid the multiple measurands coupling for the first time in addition to having a large coding capacity for wireless sensor networks in Table 1.

## 5. Conclusions

In this paper, a novel impedance-loaded OFC SAW sensor which is feasible for large-capacity passive wireless sensor networks was presented. A single-value relationship between the phase shift value and the variation of the external impedance is built. The effect of the phase shift value on the orthogonality of OFC coding is analyzed. An extended matched filter algorithm is proposed to extract the phase shift value. Simulation and experimental results demonstrate the effectiveness of this sensor. This impedance-loaded OFC SAW sensor can be used not only for structural health monitoring but also for various physical, chemical and biological measurements by replacing the external sensor. This novel impedance-loaded OFC SAW sensor could provide a large capacity for passive wireless sensor networks.

## Figures and Tables

**Table 1:** Comparison of current Impedance-loaded OFC SAW sensor performance.

	Tradition OFC SAW	Impedance-Loaded OFC SAW
Type of sensor operations	limited to one application	system and application dependent only practical limit
Simultaneous identification capability	practical measured results reduce the number within 16	identifiable tags is equivalent to factorial of chip number
